# Substrate binding modes of purine and pyrimidine nucleotides to human ecto-5′-nucleotidase (CD73) and inhibition by their bisphosphonic acid derivatives

**DOI:** 10.1007/s11302-021-09802-w

**Published:** 2021-08-17

**Authors:** Emma Scaletti, Franziska U. Huschmann, Uwe Mueller, Manfred S. Weiss, Norbert Sträter

**Affiliations:** 1 Institute of Bioanalytical Chemistry, Centre for Biotechnology and Biomedicine, Leipzig University, Deutscher Platz 5, 04103 Leipzig, Germany; 2 Helmholtz-Zentrum Berlin Für Materialien Und Energie, BESSY II, Albert-Einstein-Straße 15, 12489 Berlin, Germany

**Keywords:** eN, E5NT, Crystal structure, Purinergic signalling, Drug development, Fragment screening

## Abstract

Human ecto-5-nucleotidase (CD73) is involved in purinergic signalling, which influences a diverse range of biological processes. CD73 hydrolyses AMP and is the major control point for the levels of extracellular adenosine. Inhibitors of CD73 thus block the immunosuppressive action of adenosine, a promising approach for cancer immunotherapy. Interestingly, ADP and ATP are competitive inhibitors of CD73, with the most potent small-molecule inhibitors to date being non-hydrolysable ADP analogues. While AMP is the major substrate of the enzyme, CD73 has been reported to hydrolyse other 5′-nucleoside monophosphates. Based on a fragment screening campaign at the BESSY II synchrotron, we present the binding modes of various deoxyribo- and ribonucleoside monophosphates and of four additional fragments binding to the nucleoside binding site of the open form of the enzyme. Kinetic analysis of monophosphate hydrolysis shows that ribonucleotide substrates are favoured over their deoxyribose equivalents with AMP being the best substrate. We characterised the initial step of AMP hydrolysis, the binding mode of AMP to the open conformation of CD73 and compared that to other monophosphate substrates. In addition, the inhibitory activity of various bisphosphonic acid derivatives of nucleoside diphosphates was determined. Although AMPCP remains the most potent inhibitor, replacement of the adenine base with other purines or with pyrimidines increases the K_i_ value only between twofold and sixfold. On the other hand, these nucleobases offer new opportunities to attach substituents for improved pharmacological properties.

**Supplementary Information:**

The online version contains supplementary material available at 10.1007/s11302-021-09802-w.

## Introduction

The purinergic signalling pathway is involved in a diverse range of biological processes including platelet aggregation, neurotransmission, smooth muscle contraction, immune response and inflammation as well as the control of cell proliferation, differentiation and apoptosis [1, 2]. The purinergic signalling cascade mediates the hydrolysis of ATP into ADP, AMP and finally adenosine and free phosphate. These nucleotides play important roles as extracellular signalling molecules, acting on the P2X and P2Y receptors (ATP and ADP), and the adenosine activates P1 receptors. The levels of these nucleotides and adenosine are regulated by a group of cell surface–located enzymes called ecto-nucleotidases, which include ecto-nucleotide triphosphate diphosphohydrolases and ecto-5′-nucleotidase [3–7]. Human ecto-5′-nucleotidase (CD73, also known as eN or e5NT, gene NT5E, E.C. 3.1.3.5, UniProt P21589) is responsible for the hydrolysis of AMP and plays a critical role in switching on adenosine signalling via the P1 receptors [8]. As CD73 is the major enzyme fulfilling this role, the protein acts as a control point for the levels of extracellular adenosine. Due to this critical function, specific inhibitors targeted against CD73 have potential therapeutic applications for the treatment of chronic pain [9], inflammation [10, 11] or hypoxia [12, 13]. However, most attention for CD73 is currently based on its role as an immune checkpoint in cancer [14–19]. In the tumour microenvironment, high concentrations of extracellular adenosine promote tumour proliferation through various immunosuppressive mechanism. Many tumours overexpress CD73 and/or CD39. Inhibition of CD73 blocks the production of immunosuppressive adenosine, and this has been recognised as a promising strategy in cancer immunotherapy.

CD73 is a non-covalent homodimer, which is attached via a glycosylphosphatidyl-inositol (GPI) anchor to the extracellular membrane [20]. The structure of CD73 has been determined [21, 22] and is highly similar to bacterial 5′-nucleotidases such as *Escherichia coli* 5′-nucleotidase [23, 24]. However, in contrast to CD73, the bacterial homologue is monomeric in solution and has a wide substrate specificity. The CD73 monomer consists of two distinct domains. The N-terminal domain (residues 26–317) contains the metal-binding site, which can coordinate two zinc ions, whereas the C-terminal domain (residues 337–549) houses the substrate binding site. These two domains are linked by an α-helix (residues 318–336). In the available crystal structures, CD73 has been characterised in two distinct conformations [21].

**Fig. 1:**
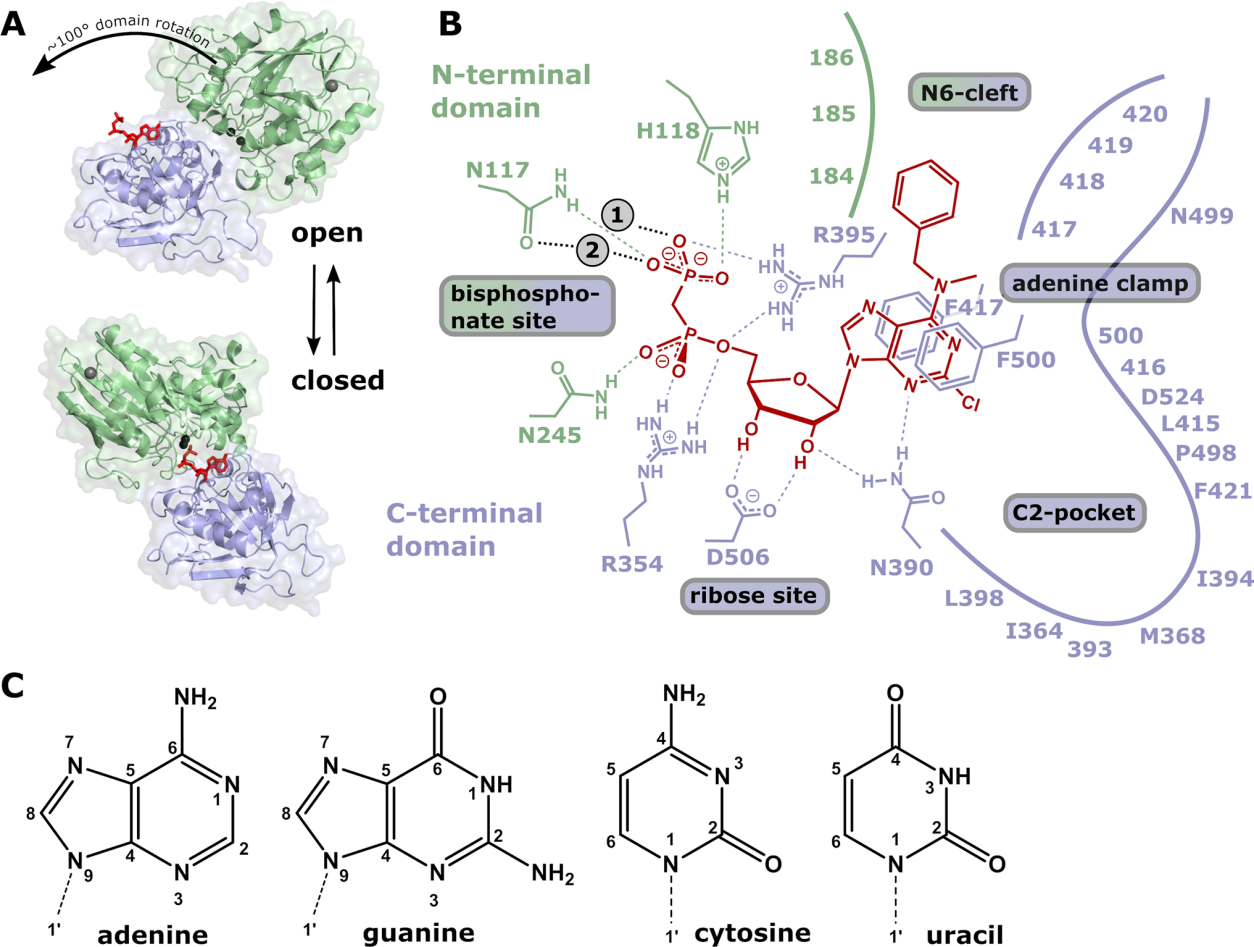
Inhibitor and substrate binding to the open and closed conformations of CD73. **A** Open (pdb id 6tvg, [36]) and closed (4h2i, [21]) conformations of CD73. The substrate binding site of the C-terminal domain is marked by bound AMPCP (red). The two conformations are shown in identical orientations of the C-terminal domain. The N-terminal domain slides along the domain interface in a ~ 100° domain rotation and positions the two catalytic zinc ions (black) and further catalytic residues into proximity of the terminal phosphate group of the substrate. **B** Scheme of the binding mode of the AMPCP derivative PSB12489 (6s7h, [35]) to the closed form of CD73. Shown are the residues that directly interact with the inhibitor. Residues forming the C2-pocket and the N6-cleft are marked with residue type and number if the side chain is involved in the formation of these structures; otherwise, only the residue number is shown. **C** Structures and atom numbering of purine and pyrimidine nucleobases

Substrate binding occurs in the inactive ‘open’ conformation where the substrate binding site of the C-terminal domain is easily accessible. The ‘closed’ conformation represents the active state where substrate hydrolysis occurs. This conformation is achieved by a large domain rotation of up to 114°, which brings the N- and C-terminal domains together forming the active site (Fig. 1). Here, the zinc ions and residues of the N-terminal domain coordinate the phosphates of the nucleotide and the C-terminal domain binds the nucleobase and ribose moiety of the substrate [21]. After hydrolysis, the product is released from the open form of CD73, as the active site is buried and poorly accessible in the closed state. The importance and characteristics of the domain motion of 5′-nucleotidase have been characterised in more detail for the *E. coli* homologue [25–28].

AMP is the major physiological substrate of CD73, with *K*_m_ values in the low micromolar range (1–50 μM [8], 7 μM [21], 59 μM [29]). CD73 has also been reported to hydrolyse other 5′-nucleoside monophosphates [3, 30]; however, detailed kinetic parameters for these substrates relative to AMP are not reported in the literature. CD73 can also utilise nicotinamide mononucleotide (110 nmol/min∙mg) and NAD^+^ (5 nmol/min∙mg) as substrates, but at much lower turnover rates compared to AMP (856 nmol/min∙mg) [31]. Notably, ADP (K_i_ = 82 nM [32], 0.91 μM [33], 3.88 μM [29]) and ATP (K_i_ = 1.5 μM [32], 8.9 μM [33]) are competitive inhibitors of CD73. It has been suggested that inhibition of CD73 by released ATP enhances the immunostimulatory action of ATP by reducing the immunosuppressive adenosine concentration in early stages of inflammation [11].

Inhibitor development for CD73 is most advanced for therapeutic antibodies, with more than 20 clinical trials currently running on four different monoclonal antibodies [16]. Small-molecule inhibitors can be advantageous in terms of the costs of a therapy and the ease of application, if they are orally available. An important starting point of many small-molecule inhibitors in current development is the analogues of the natural inhibitor ADP. α,β-Methylene-ADP (AMPCP), a commercially available non-hydrolysable analogue of ADP, inhibits human CD73 with a K_i_ of 88 nM [34]. The inhibitory potency of AMPCP is further increased by hydrophobic substituents at the N6 of the adenine base (K_i_ = 2 nM for a benzyl substituent) [34] and by a chloro substituent at C2 (K_i_ = 6 nM) [35, 36] (Fig. 1B). Many different derivatives of AMPCP have been developed independently as CD73 inhibitors, mostly in pharmaceutical companies, almost all utilising the C2-chlorine and at least one larger hydrophobic N6-substituent for improved affinity [37]. Among these is AB680 (K_i_ = 5 pM), developed by Arcus Biosciences, which has entered clinical trials [37, 38]. In addition, the inner structure of the adenine ring, the ribose or the bisphosphonate group were varied. While the acidic bisphosphonate group is expected to confer poor oral availability, it has been indicated that an oral formulation for AB680 is possible [37, 38]. Apart from non-nucleotide compounds, which are also in development [39], a replacement of the purine ring system of adenine by other nucleobases, in particular pyrimidines, is an attractive strategy to further widen the structural repertoire for CD73 inhibitor development. Junker et al. [40] recently demonstrated 4–10 nM activity for pyrimidine-based diphosphonate inhibitors.

Here, we present a comprehensive structural and kinetic analysis of CD73 inhibition by pyrimidine and purine nucleotides as well as catalytic turnover of the respective mononucleotides. The crystallographic work is based on a fragment screening campaign using 102 compounds of a HZB Bessy fragment library [41]. Using crystal soaking, high-resolution crystal structures of CD73 in the open state in complex with AMP, GMP, CMP, dCMP, UMP and IMP were obtained. Structural analysis demonstrates similar binding modes and provides insights as to why ribonucleotides are preferred by CD73. A kinetic comparison of CD73 with several commercially available non-hydrolysable nucleotide diphosphonate inhibitors is also presented, all of which exhibited IC_50_ values in the low micromolar range. While AMPCP is the most effective of the compared compounds, the inhibitory activity of other nucleobases is comparable. Most importantly, they offer new geometric possibilities to attach substituents to utilise the ‘C2-pocket’ and ‘N6-cleft’ (Fig. 1B) for designing potent CD73 inhibitors, which might lack the bisphosphonate group and have good oral availability.

## Methods

### Expression, refolding and purification

For crystallisation of CD73 in the open form, a construct was used that corresponds to the mature human CD73 protein sequence (residues 27–549) and contains the natural T376A variation (database entry P21589/VAR_022091 of UniProtKB/Swiss-Prot), four asparagine to aspartate mutations at the natural glycosylation sites, the three mutations K145S/K147S/K478S to facilitate crystallisation in the open form and a C-terminal His_6_ tag. Details of the protein production procedure have been described previously [42]. In brief, the protein was over-expressed as inclusion bodies (IBs) in *E. coli* BL21(DE3) using a pET45b( +) vector. Following expression, the cell pellet was collected by centrifugation after which the cells were lysed by sonication. Following lysis, IBs were isolated by centrifugation and resuspended in wash buffer (500 mM NaCl, 2% (v/v) Triton X-100, 20 mM EDTA pH 8.0). The IBs were then washed again in the same buffer without Triton X-100 to remove the detergent. IBs were solubilised in 6.0 M guanidinium hydrochloride after which insoluble material was removed by centrifugation. The supernatant was syringe-filtered and loaded onto a 5-mL His-Trap column. His-tagged protein was eluted over a gradient of 20–500 mM imidazole. Denatured CD73 was then refolded via rapid dilution in buffer containing 100 mM Tris–HCl pH 8.0, 500 mM l-arginine, 10% glycerol, 2 mM GSH, 1 mM GSSG, 5 mM CaCl_2_ and 30 μM ZnCl_2_. The progress of the refold was monitored using the malachite green assay, as described later in this section. Correctly folded CD73 was separated from misfolded protein by AMP-agarose affinity chromatography followed by size-exclusion chromatography.

### Crystallisation and crystal soaking

Purified CD73 was concentrated to 7 mg/mL, and hanging drop crystallizations were set up versus 100 mM Tris pH 7.8 and 10% PEG6000, using equal amounts of protein and reservoir solution. Crystallisation experiments were carried out at 19 °C. Following crystal formation (1–2 days), the crystals were transferred to soaking solution containing reservoir solution with additional commercially available nucleotides or fragments (see Table S1) at concentrations between 2 and 100 mM. Crystals were then transferred to cryo solution containing an additional 20% glycerol, soaked for ~ 2–5 min and flash frozen in liquid nitrogen.

### Data collection, structure determination and refinement

X-ray data collection was carried out at 100 K using a wavelength of 0.91841 Å on beamline 14.1 of the Berlin Synchrotron (BESSY, Berlin, Germany) equipped with a PILATUS 6 M detector [43]. All datasets were indexed, integrated, scaled and converted to structure factor amplitudes using *XDSAPP* [44] and *AIMLESS* [45]. Crystallographic statistics are presented in Tables 1 and S2. The crystals belong to crystal form II of CD73 (pdb id 4h1y), which was used as a starting model for refinement [21]. *Coot* [46] and *REFMAC5* [47] were used for model building and refinement. The final structures were validated using *PROCHECK* [48] and outliers in the Ramachandran plot were checked manually.

**Table 1:** Summary of crystallographic data statistics

Ligand	PDB-Id	d_min_ (Å)	CC_1/2_*	R_work_/R_free_ (%)	B_protein_ (Å^2^)
AMP	7P9N	1.55	0.82	15.3/17.8	10.9
GMP	7P9R	1.41	0.55	13.0/16.9	13.5
dCMP	7P9T	1.79	0.52	18.5/21.7	17.4
CMP	7PA4	1.45	0.60	11.9/16.1	13.8
UMP	7PB5	1.28	0.59	12.5/15.7	13.0
IMP	7PBA	1.42	0.56	12.7/16.7	13.2
Caffeine	7PBB	1.47	0.35	17.1/19.2	12.6
4-Nitrocatechol	7PBY	1.13	0.58	12.8/14.3	14.3
5-Iodouracil	7PCP	1.38	0.53	12.9/17.3	17.5
Riboflavin	7PD9	1.39	0.70	12.0/14.4	15.8

^*^CC1/2 in highest resolution shell. See Table S2 for further details

### Enzyme kinetics, substrate specificity and inhibition studies

In this study, enzyme activity and inhibition data was characterised using a modified malachite green assay [49, 50]. Each assay consisted of reaction buffer A (50 mM Tris–HCl pH 7.5, 100 mM NaCl, 1 mM MgCl_2_) to which the appropriate substrate and/or inhibitors (see Table S1) were added. The purity of the compounds as specified by the supplier is listed in Table S1. Dissolved compounds were immediately used for the inhibition assays. The reactions were generally initiated by the addition of protein. The protein concentrations, substrates and inhibitors used for the various experiments are detailed below. In all experiments, reactions were monitored over a 10-min period during which samples were taken at regular intervals. At each desired time point, a malachite green/molybdate (1:1) solution was added to the sample, which simultaneously terminated the assay and also allowed the detection of the produced phosphate ions. These reaction mixtures were incubated at 25 °C for 25 min, followed by measurement of the absorbance at 620 nm. A standard curve (0–50 μM orthophosphate) was performed prior to each assay and was used to calculate the phosphate concentration. All measurements were carried out in triplicate.

During protein refolding, 5 ng of protein was added to reaction buffer A containing an additional 100 μM AMP. The specific activity (μmol P_i_/min/mg protein) was calculated from the initial reaction rates and was used to determine when the protein refolding process had reached completion. For the determination for the kinetic parameters *K*_m_ and *V*_max_ for purified CD73 with the enzymes’ favoured substrate AMP, reaction buffer A was supplemented with several different substrate concentrations (5, 10, 20, 30, 40, 50, 75, 100, 150 and 200 μM AMP). Reactions were initiated by the addition of 1.25 ng of CD73. Kinetic values were determined by fitting the Michaelis–Menten equation to the initial reaction rates using GraphPad Prism (GraphPad Software, La Jolla, CA, USA). To investigate the substrate selectivity of human CD73, enzyme activity was tested against a panel of commercially available nucleotides (AMP, dAMP, GMP, dGMP, CMP, dCMP, dTMP, UMP and IMP; see Table S1). In these tests, reaction buffer A was supplemented with 1 mM of the desired substrate and reactions were started with 1.25 ng of CD73. The specific activity (μmoles P_i_/min/mg protein) was calculated for each substrate, and the percentage activity was then calculated relative to AMP (100% activity).

To study the inhibition kinetics of CD73 against a panel of commercially available nucleotide-diphosphate analogues (AMPCP, GMPCP, dUMPCP and CMPCP; see Table S1), their IC_50_ values were determined. In these tests, CD73 protein (1.25 ng) was incubated in reaction buffer A containing various inhibitor concentrations (0.015, 0.031, 0.061, 0.12, 0.24, 0.49, 0.98, 1.95, 3.91, 7.81, 15.62, 31.25, 62.5, 125, 250 and 500 μM). Following an incubation period of 5 min, the reactions were then initiated by the addition of 100 μM AMP. Dose–response curves were plotted in GraphPad Prism (GraphPad Prism Software, La Jolla, CA, USA), and the inflection point of each curve was used to determine the IC_50_ value of each inhibitor. The IC_50_ values were then used to calculate the *K*_i_ value of each inhibitor using the Cheng-Prusoff equation [51].

## Results

### Fragment screening

The HZB fragment library used for this work contains 102 compounds [41]. Of these, 24 hits (defined binding poses) were obtained for 22 fragments: 16 compounds did bind to the nucleoside binding site (12 nucleotides and 4 non-nucleotides; Table S3), 6 compounds bound at crystal contacts and 2 compounds bound at the interface between the two domains. The presence of 12 compounds containing a nucleoside moiety in the fragment library contributed to the high hit rate of 23.5%.

### Binding mode of the natural substrate AMP to the open form of CD73

**Fig. 2:**
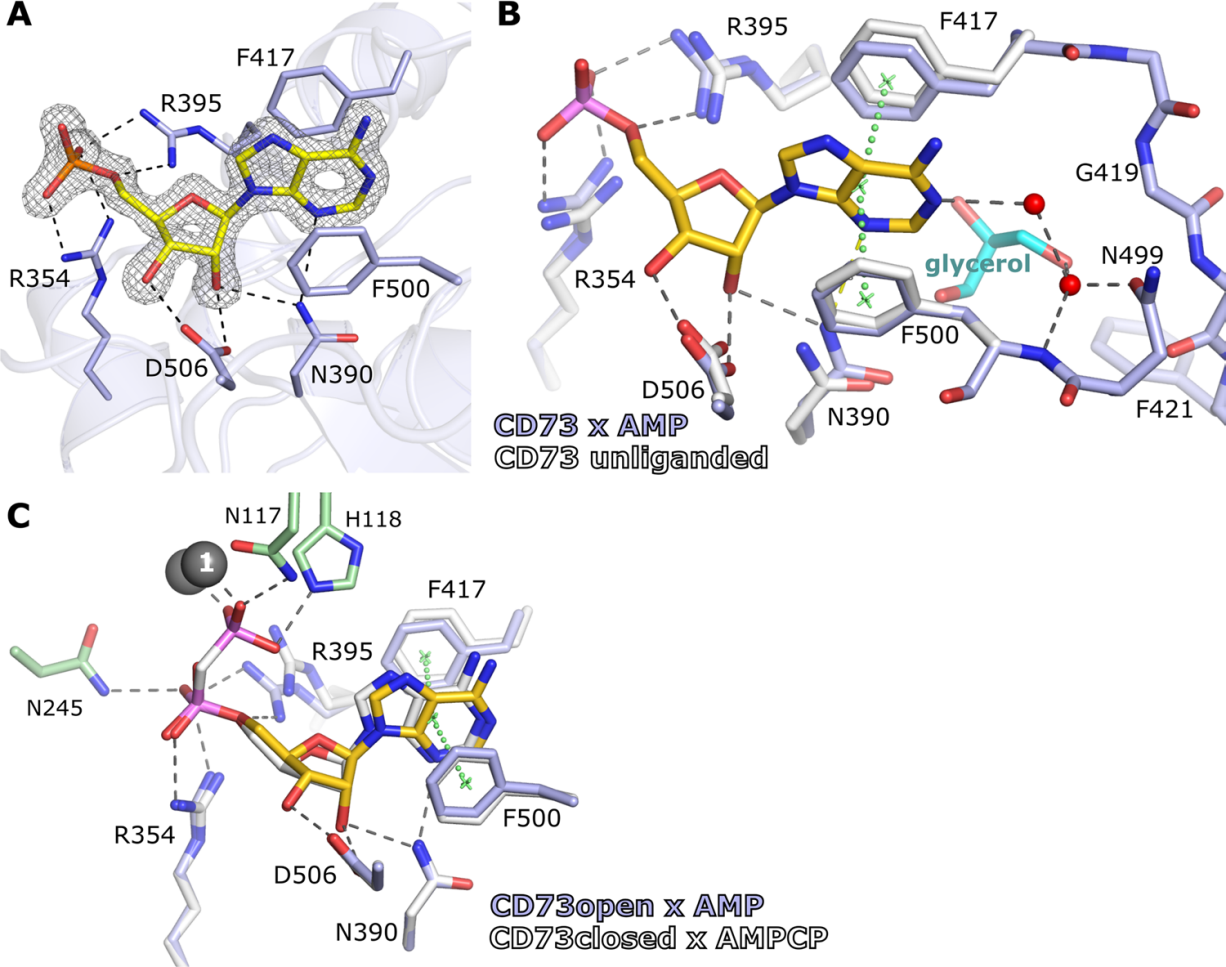
Recognition of AMP by CD73. **A** Substrate binding mode of AMP (yellow) to the open state of CD73. The (2*F*_o_–*F*_c_)-type electron density map around the AMP substrate is contoured at 1.5 σ. **B** Superposition of CD73^open^ × AMP with unliganded CD73 (pdb id 6tve) in the open form. **C** Superposition of CD73^open^ × AMP with CD73^closed^ × AMPCP (pdb id 4h2i). Residues of the N-terminal domain of CD73^open^ × AMP are coloured in green whereas those of the C-terminal domain are depicted in blue. The superimposed molecules are shown in white

The structure of CD73 in complex with AMP was determined to 1.69 Å resolution (Table 1), and the AMP substrate is well defined by the electron density (Fig. 2A) indicating full occupancy and no flexibility. The adenosine base is positioned by a π-stacking interaction with Phe417 and Phe500. There are no amino acid side chains within hydrogen bonding distance to the adenine ring, except for the interaction of N3 with the NH_2_-group of Asn390 at a rather long distance of 3.3 Å. The N1 of adenine interacts with the protein via two water molecules (Fig. 2B). The ribose group of AMP is positioned by hydrogen bonds with Asn390 and Asp506, whereas the phosphate group is coordinated by Arg354 and Arg395 (Fig. 2A).

### Comparison of the AMP-bound open form with the unliganded enzyme and the closed form

A comparison of the CD73^open^ × AMP structure with unliganded CD73 in the same crystal form (pdb id 6tve, [36]) shows only minor movements of the side chains of Arg354, Arg395 and Phe417 in response to substrate binding (Fig. 2B). These residues maintain the same conformation, and the active site structure is thus preformed for AMP binding. An exception is Phe500, which exists in two conformations in the unliganded enzyme and the conformation with occupancy 0.36 disappears in the nucleotide-bound structure due to steric overlap with the adenine ring.

An interesting feature relevant for inhibitor development is the large pocket next to the C2 atom of the adenine ring (‘C2-pocket’, Fig. 1). This pocket is filled with water molecules. Interestingly, a glycerol molecule from the cryo buffer has replaced three water molecules from the C2-pocket and the hydroxyl groups of the glycerol form favourable interactions with the polar rim of the pocket (Fig. 2B). This glycerol molecule is an interesting fragment for connection to the C2 of the adenine ring (or other inhibitor scaffolds stacked between Phe417 and Phe500), in particular if small hydrophobic substituents could be attached to the central secondary carbon atom of the glycerol group to interact with the hydrophobic base of the C2-pocket.

Comparison of CD73^open^ × AMP with CD73^closed^ × AMPCP (pdb id 4h2i, [21]) shows that the adenine base and ribose moiety of the two nucleotides superimpose very well (Fig. 2C). The α-phosphates from AMP and AMPCP also have very similar binding modes. However, in the closed form, the α-phosphate makes additional interactions with Asn245 and His243 from the N-terminal domain. Possibly, no structural rearrangements of active site residues are necessary to form the catalytically competent AMP binding mode for catalysis. However, the AMP binding mode of CD73 in the closed form has not been structurally characterised yet. In addition, concerning inhibitor development, it is evident, due to the optimal superposition of AMP and AMPCP between the open and closed form structures, that the open form is a suitable means for studying the binding of monophosphate nucleotides other than AMP to CD73.

### Nucleotide substrate preferences of CD73

**Fig. 3:**
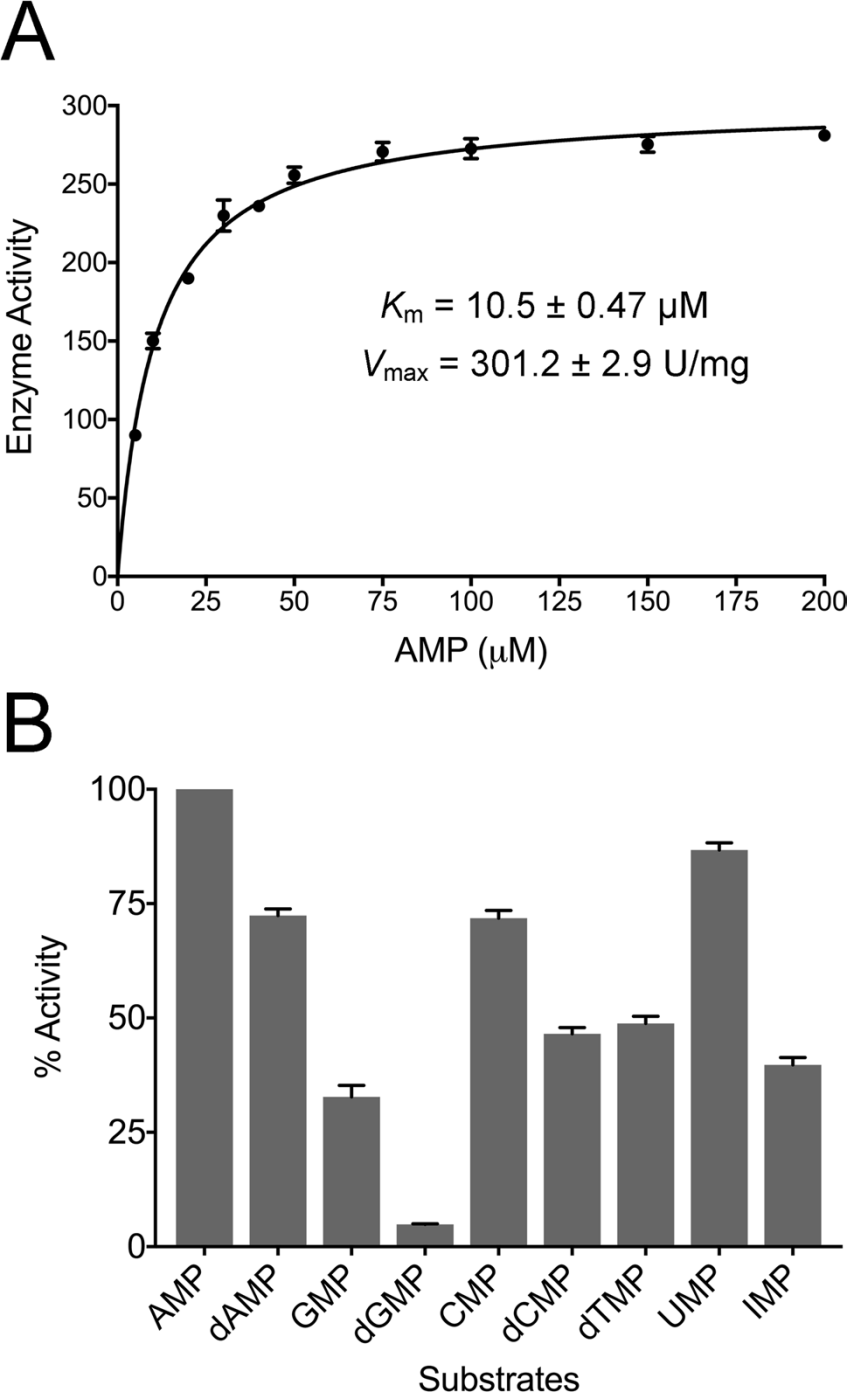
Kinetic analysis of CD73 with various nucleotide substrates. In all experiments, substrate hydrolysis was measured using the malachite green assay and initial rates of reaction were determined in triplicate. **A** Michaelis–Menten kinetics for CD73-mediated hydrolysis of AMP. CD73 was incubated with concentrations of AMP ranging from 0 to 200 µM. Data are presented as specific activity (μmol Pi/min/mg protein) versus AMP (µM). **B** Comparison of substrate preferences for CD73 against a panel of commercially available purine and pyrimidine nucleotides. The specific activity (μmol Pi/min/mg protein) of CD73 was calculated at a concentration of 1 mM for each substrate. The percentage activities of dAMP, GMP, dGMP, CMP, dCMP, dTMP and UMP are all calculated relative to the favoured substrate AMP where the percentage activity is set as 100%

Prior to characterisation of the binding modes of various nucleoside monophosphates, the substrate preference was characterised (Fig. 3). CD73 has a *K*_m_ for AMP of 10.5 μM and a *V*_max_ of 301.2 U/mg. This specific activity is higher compared to human CD73 purified from an adenocarcinoma cell line (130 U/mg) [52], but lower than the value determined previously for this construct after refolding (577.2 U/mg) [21]. The *K*_m_ value, however, is very similar to the value reported from the aforementioned study (6.6 μM) [21], and it is also comparable to the values described for human CD73 obtained from other sources (1–50 μM) [8, 53].

In order to investigate the substrate selectivity of CD73, enzyme activity was tested against a variety of ribonucleotide monophosphates and their corresponding deoxyribonucleotide forms (AMP, dAMP, GMP, dGMP, CMP, dCMP, dTMP, UMP and IMP). In these tests, the percentage activity of each substrate was calculated relative to the activity of AMP, which was set as 100% (Fig. 3B). CD73 displayed a clear preference for ribonucleotide monophosphates over their equivalent deoxyribose forms. This equated to a decrease in absolute activity (relative to AMP) of 28.8% for dAMP compared with AMP, 28.4% for dGMP compared to GMP and a 25.5% decrease for dCMP compared to CMP. Comparison of AMP with other ribonucleotide monophosphate substrates indicated that CD73 had the lowest activity with the purines GMP and IMP (33.2% and 40.3%). Interestingly, the enzyme activity was more similar to the pyrimidine substrates UMP and CMP (87.1% and 73.4% respectively). This trend was also observed when analysing dAMP against other deoxyribonucleotide monophosphate substrates. CD73 displayed the lowest relative activity with the purine dGMP (4.8%) but was more active against the pyrimidine deoxynucleotide substrates dCMP and dTMP (47.6 and 49.4%, respectively).

### Binding mode of other nucleoside monophosphate substrates

**Fig. 4:**
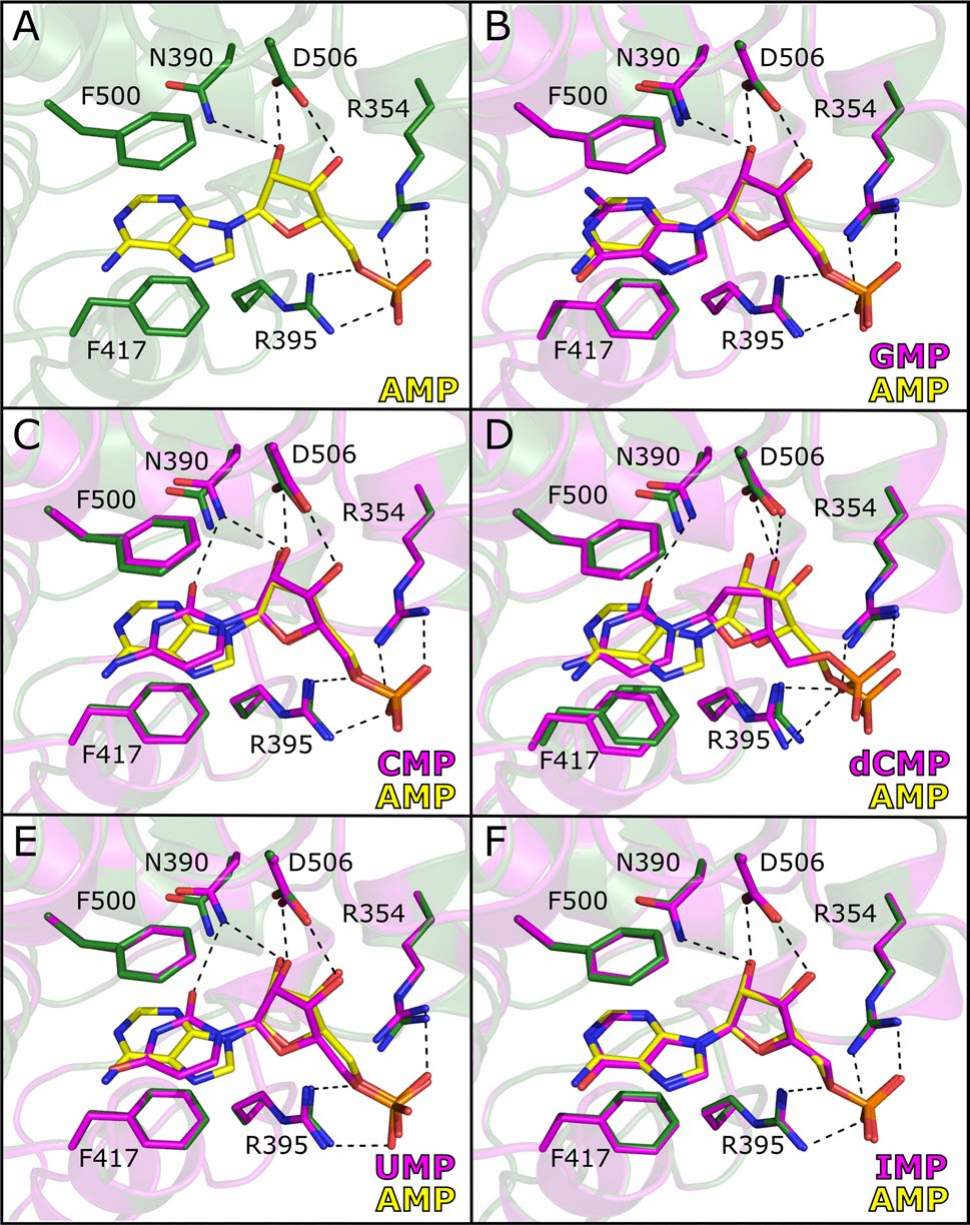
Comparison of the AMP binding mode with other nucleotide substrates. **A** Hydrogen bonding interactions of CD73 with AMP (yellow). Superpositions of GMP (**B**), CMP (**C**), dCMP (**D**), UMP (**E**) and IMP (**F**) are also shown. Throughout panels **A**–**E**, amino acids contributing to ligand binding are depicted as sticks. Carbon atoms are coloured green (AMP-bound CD73) or magenta (structures bound with other nucleotides). The hydrogen bond between Asp506 and Asn390 marked in blue dashes in panel **D** is only present in the CD73 × dCMP structure, most likely to compensate for the loss of the 2′-OH group as a hydrogen bonding partner for Asp506

In order to study the structural basis of selectivity for nucleoside monophosphate substrates, structures of CD73 were determined in the open state in complex with GMP, CMP, dCMP, UMP and IMP (Fig. 4). As noted previously, this involved soaking open form crystals as these substrates would be hydrolysed in co-crystallisation experiments for the closed form. The high-resolution structures (1.28–1.79 Å) (Table 1) obtained all contained unambiguous density for the substrates in the C-terminal nucleotide binding domain. There was clear density for the phosphate group of all nucleoside monophosphate structures, as is depicted for AMP in Fig. 2A.

Superposition of CD73^open^ × AMP with the corresponding complex structures of GMP, CMP, dCMP, UMP and IMP shows the nucleotides to generally superimpose well (Fig. 4). The nucleobase of the purine nucleotides GMP (Fig. 4B) and IMP (Fig. 4F) match particularly well, positioned by a π-stacking interaction between Phe417 and Phe500 (Fig. 4A). The nucleobase moieties of the pyrimidine nucleotides CMP, dCMP and UMP (Fig. 4C–E) position themselves centrally between the two conserved phenylalanine residues. In comparison to the adenine ring of AMP, the pyrimidine bases are located in plane and close to the centre between the two ring systems of the adenine group. Compared to AMP, the pyrimidine nucleotides CMP and UMP can position the C2 carbonyl oxygens closer to the NH_2_-group of Asn390, and thereby form strong hydrogen bonding interactions. In addition, the pyrimidine bases of CMP and UMP interact via two water molecules with the water network of the C2-pocket. In conclusion, the pyrimidine bases are well connected to the water network of the C2-pocket. They also position the N4 (CMP) or O4 (UMP) atoms close to the N6 atom of the adenine moiety of AMP, such that the addition of similar hydrophobic substituents is possible to utilise the affinity gained by interaction with the N6-cleft. However, the direction of the C4-X4 (X4 = N,O) bond differs by ~ 35° from the C6-N6 bond in the AMPCP derivatives, meaning that the use of pyrimidines offers new opportunities for derivatization.

Comparison of the ribose groups of the ribonucleoside monophosphate substrates (GMP, CMP, UMP and IMP) with AMP shows conserved binding modes and interactions. Comparison of the complex structures with AMP and dCMP, however, shows significant differences at this position (Fig. 4D). Most significantly, the ribofuranoses adopt a C3′-*endo* conformation concerning the sugar pucker, whereas the 2′-desoxy-ribofuranose in dCMP has a C2′-*endo* conformation. In the dCMP complex, the lack of the 2′-OH group also results in the loss of two hydrogen bonds compared to the AMP structure. This results in a shift of the whole nucleotide structure by about 0.8 Å compared to AMP and the other substrates with a 2′-OH group. Also, the phosphoester linkage of dCMP adopts a different conformation compared to the other structures.

### Binding mode of additional fragments to the active site

**Fig. 5:**
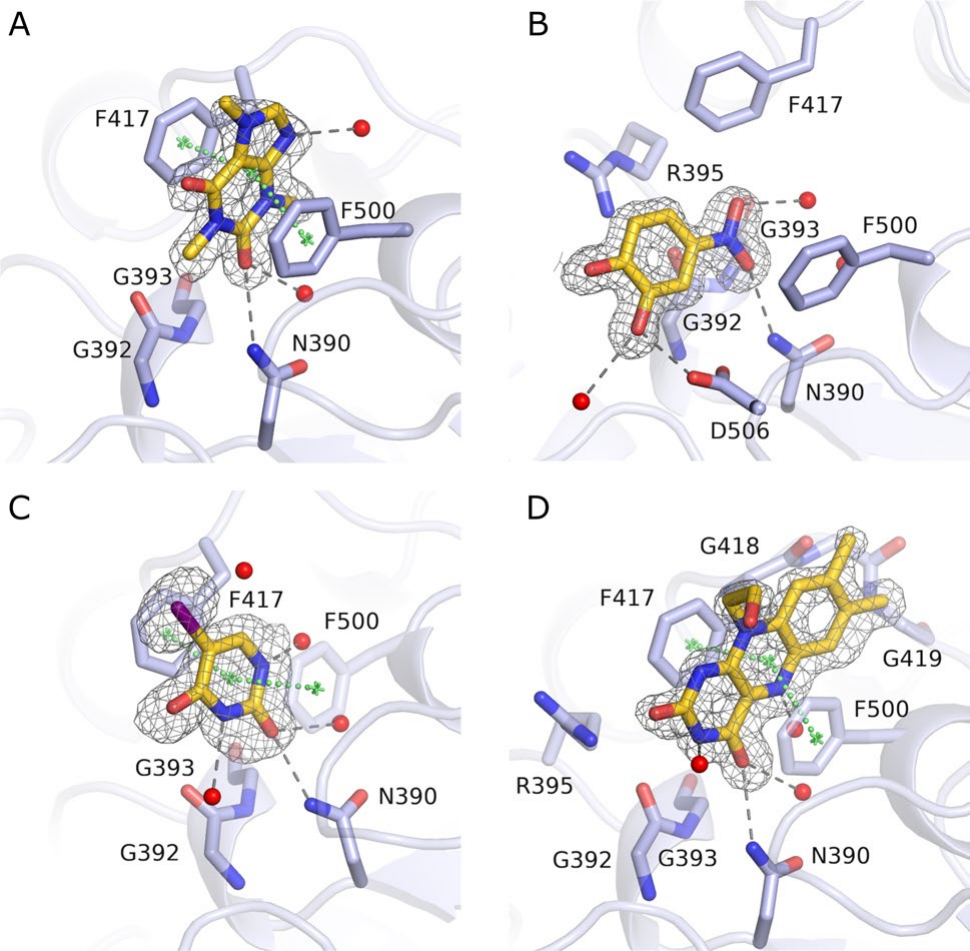
Binding modes of four fragments binding to the nucleoside binding site of CD73. Shown are the binding modes of **A** caffeine, **B** 4-nitrocatechol, **C** 5-iodouracil and **D** riboflavin. The polder omit electron density map [55] is shown at a contour level of 5 σ_rms_. In panels **C** and **D**, Phe500 is depicted with transparency for a better view of other ligand interactions

In addition to the 12 compounds containing a nucleoside moiety, four additional fragments of the HZB fragment screen [41] did bind to the nucleoside binding site: caffeine (Fig. 5A), 4-nitrocatechol (Fig. 5B), 5-iodouracil (Fig. 5C) and riboflavin (Fig. 5D). The dominant interaction for the binding of caffeine, 5-iodouracil and riboflavin is the π-stacking between Phe417 and Phe500 and a hydrogen bonding interaction of an oxo group of the inhibitors with Asn390 (Fig. 5 and S1). Caffeine and flavin share a similar six-membered ring structure with uracil, and the uracil ring system is oriented in a similar manner (Fig. S1). The electron density maps are well defined, indicating a single low-energy binding mode. The only exception is iodouracil, where different refinement trials indicated that the iodine position is not fully occupied. This may result from additional multiple binding modes that could not be clearly identified by the electron density maps or from radiation damage leading to partial loss of the iodine atom.

Despite its planar aromatic structure, 4-nitrocatechol does not bind in the adenine clamp of Phe417 and Phe500, but at the ribose binding site (Fig. 5B). It interacts via one of the two phenolic OH groups with Asp506. This residue forms hydrogen bonds to both hydroxyl groups of the ribose group in bound nucleosides (Fig. 4). Nevertheless, Asn390 also plays a role in binding this compound by forming a hydrogen bond to the nitro group.

### Inhibition of CD73 by phosphonic acid analogues of nucleoside diphosphates

**Fig. 6:**
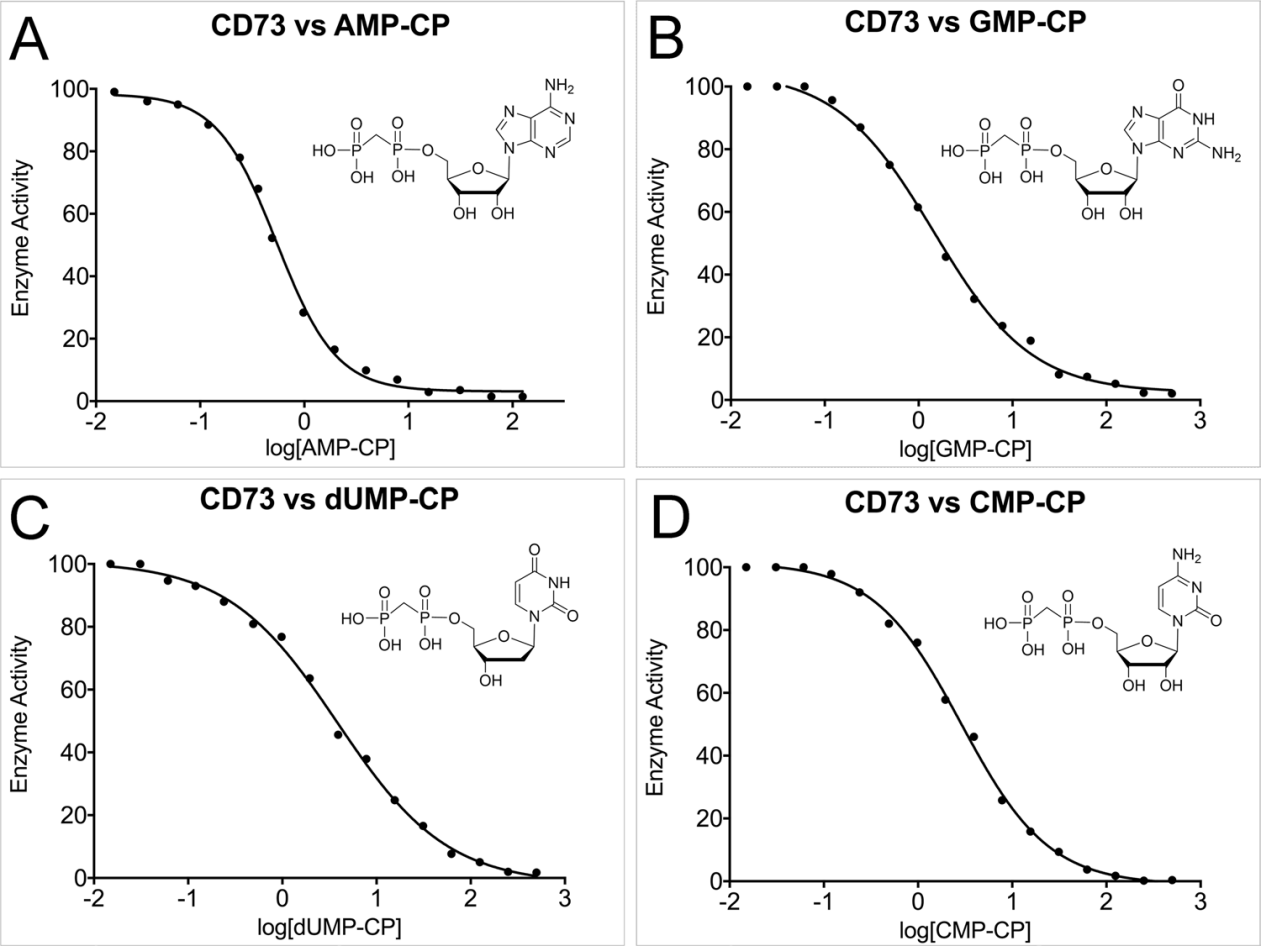
Inhibition kinetics of CD73 against a panel of commercially available nucleotide-diphosphate analogues. Dose–response curves of human CD73 against AMPCP (**A**), GMPCP (**B**), dUMPCP (**C**) and CMPCP (**D**). [compound concentration], *y*-axis: percentage inhibition of CD73. In all experiments, substrate hydrolysis was measured using the malachite green assay and initial rates of reaction were determined in triplicate. The inflection point of each curve was used to determine the IC_50_ value of each compound, which was carried out in GraphPad Prism (GraphPad Software, La Jolla, CA, USA)

Due to the ability of CD73 to hydrolyse multiple nucleoside monophosphate substrates (Fig. 3B), we decided to assess the potential of phosphonic acid analogues of nucleoside diphosphates other than AMPCP as CD73 inhibitors (Fig. 6). IC_50_ values were obtained for the commercially available compounds AMPCP, GMPCP, CMPCP and dUMPCP, which 7PD9 were then converted to *K*_i_ values using the Cheng-Prusoff equation [51]. For the compounds tested against CD73, AMPCP displayed the highest potency (IC_50_ = 0.56 ± 0.01 μM, *K*_i_ = 59 ± 1.5 nM). In terms of the other commercially available compounds, GMPCP was the second most potent compound (IC_50_ = 1.42 ± 0.02 μM, *K*_i_ = 135 ± 2.7 nM). The two pyrimidine-based nucleoside bisphosphonates analysed had lower affinity against CD73 than the purines AMPCP and GMPCP. The best of these compounds was CMPCP (IC_50_ = 3.20 ± 0.02 μM, *K*_i_ = 304 ± 5.8 nM) followed by dUMPCP (IC_50_ = 3.94 ± 0.01 μM, *K*_i_ = 374 ± 6.9 nM). Overall, the loss in affinity between the best and worst of these substrate analogues compared to AMPCP ranged from two- to sixfold.

## Discussion

CD73 is the major enzyme responsible for AMP hydrolysis in the cell, and thereby represents a control point for the levels of extracellular adenosine. Inhibitors of CD73 therefore have potential therapeutic applications for treating chronic pain, inflammation, hypoxia and cancer. The interaction of CD73 with adenosine or AMPCP has been studied previously, including many AMPCP derivatives [21, 35, 36, 38]. However, there is a deficit of structural and kinetic data involving other nucleotide monophosphate substrates and the binding mode of the natural substrate AMP has not been determined previously. Here, we have determined high-resolution structures of CD73 in complex AMP, GMP, dCMP, CMP, UMP and IMP and performed substrate screening with a broad panel of nucleotide monophosphates. Superposition of CD73^open^ × AMP with CD73^closed^ × AMPCP showed that the base and sugar moieties superimposed very well, confirming our structures to be a suitable means of studying the binding of alternative monophosphate substrates.

For the panel of nucleotide monophosphates tested, all were worse substrates than AMP to varying extents. Interestingly, the purine nucleotide monophosphates GMP and IMP were the worst of the alternative substrates tested, despite the fact that the binding mode of these structures is very similar to AMP. The purine IMP, for example, is a considerable worse substrate than the pyrimidine UMP, even though it is significantly more structurally similar to AMP and superimposes very well in its binding mode to the open form. We hypothesise that the presence of the C6 carbonyl group in GMP and IMP is responsible for these differences in activity. In the CD73^closed^ × AMPCP structure, the nitrogen of the NH_2_ group at C6 is at a distance of 3.9 Å to the carbonyl oxygen of Leu184. Due to the shorter linker to the terminal phosphate group in AMP compared to AMPCP, the two domains have to move closer together such that the phosphate group of AMP can coordinate to the dimetal centre for hydrolysis. This conformation may result in a hydrogen bridge between the NH_2_-group at C6 and the carbonyl group of Leu184. A replacement of the amino group with an oxygen atom may destabilise this domain orientation and catalytically competent binding mode, resulting in diminished activity. However, the pyrimidine inhibitor UMP also has a carbonyl group at the corresponding position, but this compound is hydrolysed almost as efficient as AMP. Possibly, the smaller pyrimidine nucleobase has more flexibility to avoid a close interaction with Leu184.

The loss in inhibitory activity of the pyrimidine-based compound CMPCP compared to AMPCP is probably due to the less strong π-stacking interactions of the smaller ring system in the pyrimidines. Stacking interactions between aromatic compounds and nucleobases are critical for the strength and selectivity of nucleotide binding [54]. Nevertheless, the pyrimidine nucleoside monophosphates are readily hydrolysed by CD73 (Fig. 3). We assume that this is due to the assay conditions approaching substrate saturation at concentrations of 1 mM. We determined a K_M_ value of 10.5 μM for AMP hydrolysis. If we assume that the K_M_ values for the pyrimidine nucleoside monophosphates are up to tenfold higher, the substrate concentration is still tenfold higher than the K_M_ value and the specific activity is not much affected by the substrate binding affinity as long as a catalytically competent binding mode can be achieved. The latter assumption is supported by the similar binding modes of the pyrimidine and purine compounds (Fig. 4).

During substrate screening, we consistently found CD73 to have much poorer activity towards deoxyribose monophosphate substrates than their ribonucleotide monophosphate equivalents. This observation is readily explained by our structural data, where the CD73^open^ × dCMP complex indicates the loss of two hydrogen bonding interactions that are present in the ribonucleoside monophosphate complexes. The loss of these interactions noticeably affects the positioning and conformation of the sugar moiety as well as of the phosphate ester linkage (Fig. 4D). This is probably the cause for the less efficient hydrolysis of these compounds. However, concerning the use of deoxyribonucleotides in CD73 inhibition, the comparison between CMPCP with dUMPCP shows that the loss in affinity is only 1.2-fold. The fact that these two compounds have different but similar nucleobases indicates that the reduction in affinity resulting from the loss of the 2′-OH group is relatively small. This may be explained by considering that the hydrogen bonds lacking in the bound deoxyribonucleoside binding modes are also absent in the unbound structures, i.e., the 2′-OH of the ribofuranose forms favourable hydrogen bonds not only in the bound state but also in the unbound state (to water molecules) whereas these interactions are lost in the bound and unbound states of deoxyribose. The net gain in binding energy ΔΔG contributed by the 2′-OH group can thus be small, if the interacting protein residues also have energetically comparable interactions in the bound and unbound states. Asn390 forms a hydrogen bond to the Asp506 side chain in the CD73 × dCMP structure to compensate for the missing 2′-OH group as an H-bonding partner (Fig. 4D). Since the differences in nucleotide binding affect catalysis but not competitive inhibition, the deoxyribonucleotide compounds have an affinity comparable to that of the ribonucleotides.

All of the nucleoside disphosphate analogues tested in this study were effective inhibitors of CD73, with affinity differences ranging from two- to sixfold. The best inhibitor was AMPCP, consistent with AMP being the preferred and natural substrate of the enzyme. We determined a K_i_ value of 59 nM for AMPCP against our human deglycosylated CD73 construct. Previously reported values are 88.4 nM for recombinant soluble enzyme in glycosylated form and 150–200 nM for membrane-bound CD73 expressed in different cell lines [35]. AMPCP compounds are potent CD73 inhibitors which have been shown to display high selectivity against other ecto-nucleotidases and ADP-activated as well P2Y receptors [34]. Nevertheless, it may turn out that AMPCP derivatives show off-target effects considering the large number of proteins interacting with adenine nucleotides. Such off-target effects increase the risks of toxic side effects during treatment and emphasises that in addition to the improvement of these compounds, there is also a need for the identification of novel CD73 inhibitors with different structures.

In agreement with previous work [40], we have demonstrated here that other nucleobases also exhibit high inhibitory potency against CD73. We have characterised the structural basis of inhibition, which provides new possibilities for derivatization to improve pharmacological parameters. Indeed, the first studies on pyrimidine-based CD73 inhibitors with low nanomolar affinity have been recently reported [40]. For the pyrimidine nucleoside bisphosphonates, we determined K_i_ values of 304 ± 5.8 nM and 374 ± 6.9 nM for CMPCP and dUMPCP, respectively. For CMPCP and UMPCP, K_i_ values of 898 ± 63 nM and 1830 ± 530 nM were determined for rat CD73 [40]. It was found that adenine as well as pyrimidine nucleoside bisphosphonates are usually about two- to threefold more potent towards human CD73 compared to rat CD73 [34, 40]. AMPCP inhibits rat CD73 with a K_i_ of 197 ± 5.0 nM [34]. Thus, the ratio K_i_ (CMPCP)/K_i_ (AMPCP) determined for the human CD73 construct in this work is with a value of 5.1 in good agreement with the value of 4.6 determined for rat CD73.

Four additional fragment hits demonstrate the dominant role of the adenine camp (Phe417 and Phe500) and of Asn390 for inhibitor binding (Fig. 5). In particular the binding modes of caffeine or the flavin group suggest additional options to replace the purine or pyrimidine nucleobases for interaction with the adenine clamp. The larger three-ring flavin group extends the interactions with the adenine clamp away from the ribose binding site forming additional interactions with the main chain atoms of Phe417 and Phe500 and with the Gly418 and Gly419 (Figure S3). If the inhibitor would be further extended in this direction, it gets in contact with the N-terminal domain (in the open form). This may lead to the generation of inhibitors locking the enzyme in the inactive, open conformation. The high-resolution structures analysed in this work facilitate structure-based design of novel CD73 inhibitors.

## Supplementary Information

Below is the link to the electronic supplementary material.

Supplementary file1 (PDF 1.30 MB)
