# Supplementary material for: Substrate binding modes of purine and pyrimidine nucleotides to human ecto-5′-nucleotidase (CD73) and inhibition by their bisphosphonic acid derivatives
Source: Purinergic Signal. 2021 Aug 17;17(4):693–704. doi: 10.1007/s11302-021-09802-w (PMC8677862; doi:10.1007/s11302-021-09802-w)
Supplement: Supplementary file 1 — Supplementary file1 (PDF 1.30 MB) [file 11302_2021_9802_MOESM1_ESM.pdf]

Supplementary Information

Substrate binding modes of purine and pyrimidine nucleotides to human ecto-5'-nucleotidase (CD73) and inhibition by their bisphosphonic acid derivatives

Emma Scaletti[1], Franziska Huschmann[2], Uwe Mueller[2], Manfred S. Weiss[2] and Norbert Sträter[1]

[1]Institute of Bioanalytical Chemistry, Centre for Biotechnology and Biomedicine, Leipzig University, Deutscher Platz 5, 04103 Leipzig, Germany

[2]Helmholtz-Zentrum Berlin für Materialien und Energie, BESSY II, Albert-Einstein-Straße 15, 12489 Berlin, Germany

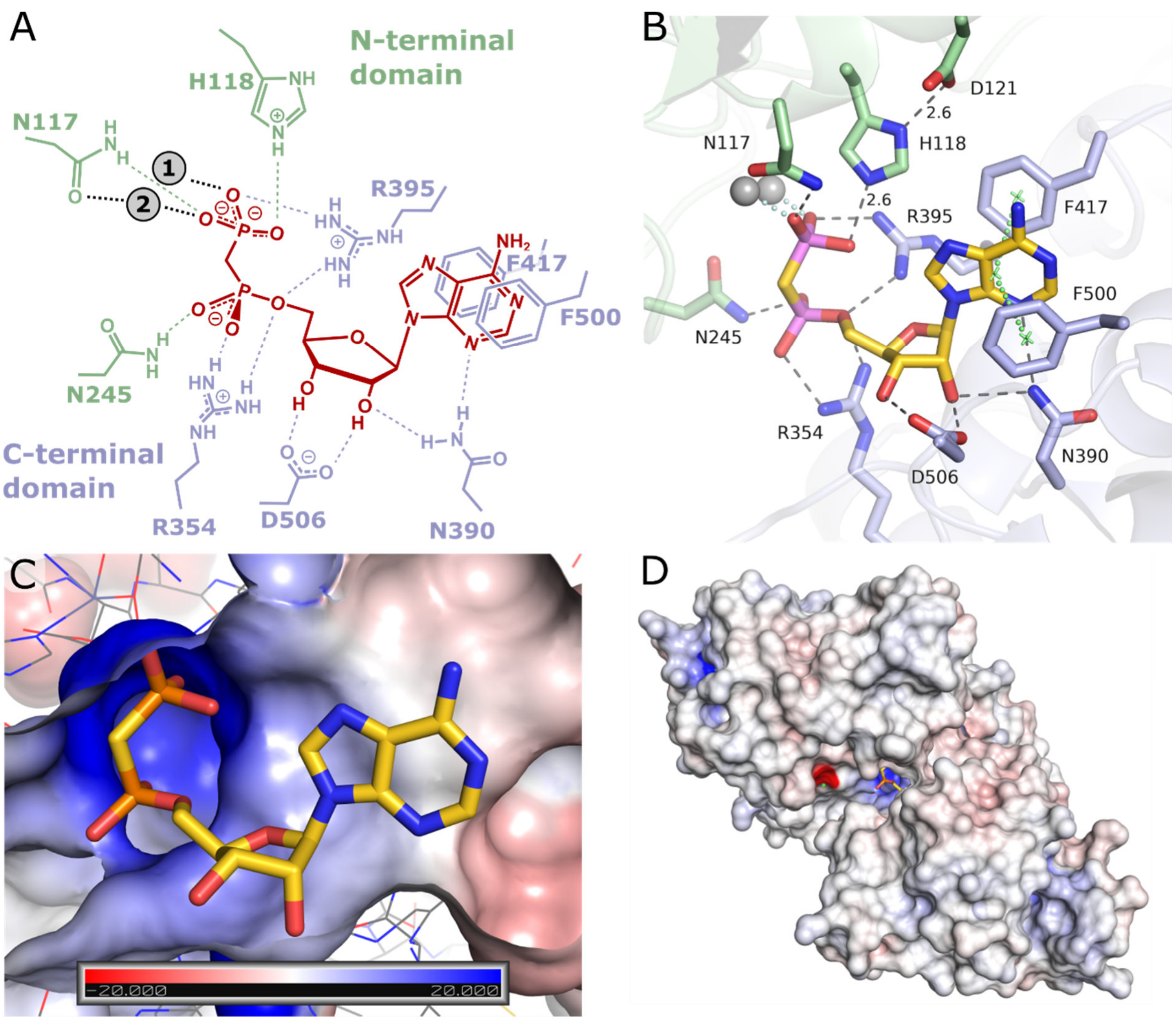

Figure S1: Possible protonation state of the active site in the CD73×AMPCP complex. (A) Scheme of the interactions of AMPCP with CD73 in the crystal structure pdbid 4h2i [1]. (B) Crystallographic model of AMPCP bound to the active site of CD73 based on the structure 4h2i. (C) Electrostatic potential of the AMPCP binding site. The electrostatic potential at the solvent accessible surface is mapped onto the Connolly surface shown in the figure. The potential is colored from -20 to +20 kT/e as indicated in the scale at the bottom of the image. APBS has been used for calculation of the potential and the ions (two Zn^{2+}, Ca^{2+} and Cl^{-}) of pdb id 4h2i have been included in the model, but not the AMPCP ligand. The

histidine ligands are not charged in this calculation. (D) Comparison to the electrostatic potential of one side of the protein calculated and depicted with the same parameters as in (C).

The protonation state suggested in scheme (A) is not based on direct experimental evidence but on the following considerations. Two of the three protons of the bisphosphonate group of AMPCP are strongly acidic, whereas the last proton on the β-phosphonate group dissociates at weakly alkaline pH with a pK_a of 8.3 [2, 3]. The pK_a value is reduced to 5.8 in the presence of coordinated Mg^{2+} ions. In CD73, the terminal phosphate group is coordinated to two divalent metal ions (most likely Zn^{2+} in the crystal structure). In addition to the zinc ions, two positively charged arginine residues are coordinated to the bisphosphonate group. As outlined below, also His118 is likely positively charged. In the absence of bound AMPCP, the binding site for the bisphosphonate group of CD73 has a strongly positive electrostatic potential of around +20 kT/e, even without including the assumed positive charge of His118 (Figure S1C). In this environment, the pK_a of the weakly ionizable proton of AMPCP is assumed to be reduced well below 7.0, such that the bisphosphonate group is probably completely deprotonated. The assumption of a positive charge on His118 is based predominantly on its polar interactions with Asp121 and AMPCP (Figure S1B). Asp121 and His118 form a strong hydrogen bonding interaction based on the N-O distance (2.6 Å) and small deviation (15°) of the N-H···O angle from colinearity. The likely negative charge of Asp121 supports protonation of His118. His118 is also perfectly positioned for a hydrogen bonding interaction with AMPCP (2.6 Å distance and only 8° deviation of the N-H···O angle from colinearity). This close interaction would be quite unfavorable without a hydrogen bonding interaction. Without consideration of the active site environment, comparison of the pK_a values of the protonated histidine (6.0) and AMPCP (8.3) would favour binding of the proton to AMPCP. However, the pK_a of the terminal AMPCP phosphonate group is likely reduced substantially, as outlined above. The hydrogen prediction tool for protein-ligand complexes PROTOSS [4] places the proton at His118, as does the Protonate3D algorithm of MOE. Both algorithms suggest the protonation state shown in Figure S1A. A protonated state of His118 is also considered as a requirement for the presumed role of this residue in protonation of the leaving group or stabilization of transition state in the catalysis of phosphate ester hydrolysis [5, 6]. The Asp-His dyad is structurally conserved in many members of the superfamily of calcineurin metallophosphatases.

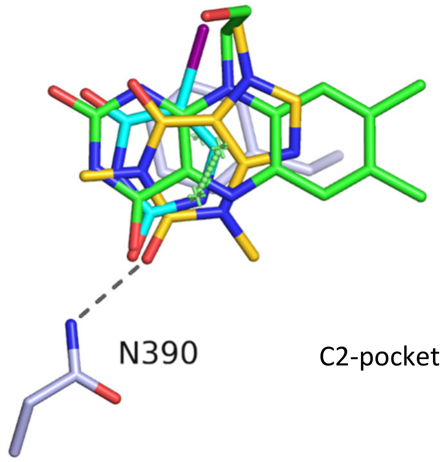

Figure S2. Superposition of the binding modes of caffeine (yellow), iodouracil (cyan) and riboflavin (green) to CD73.

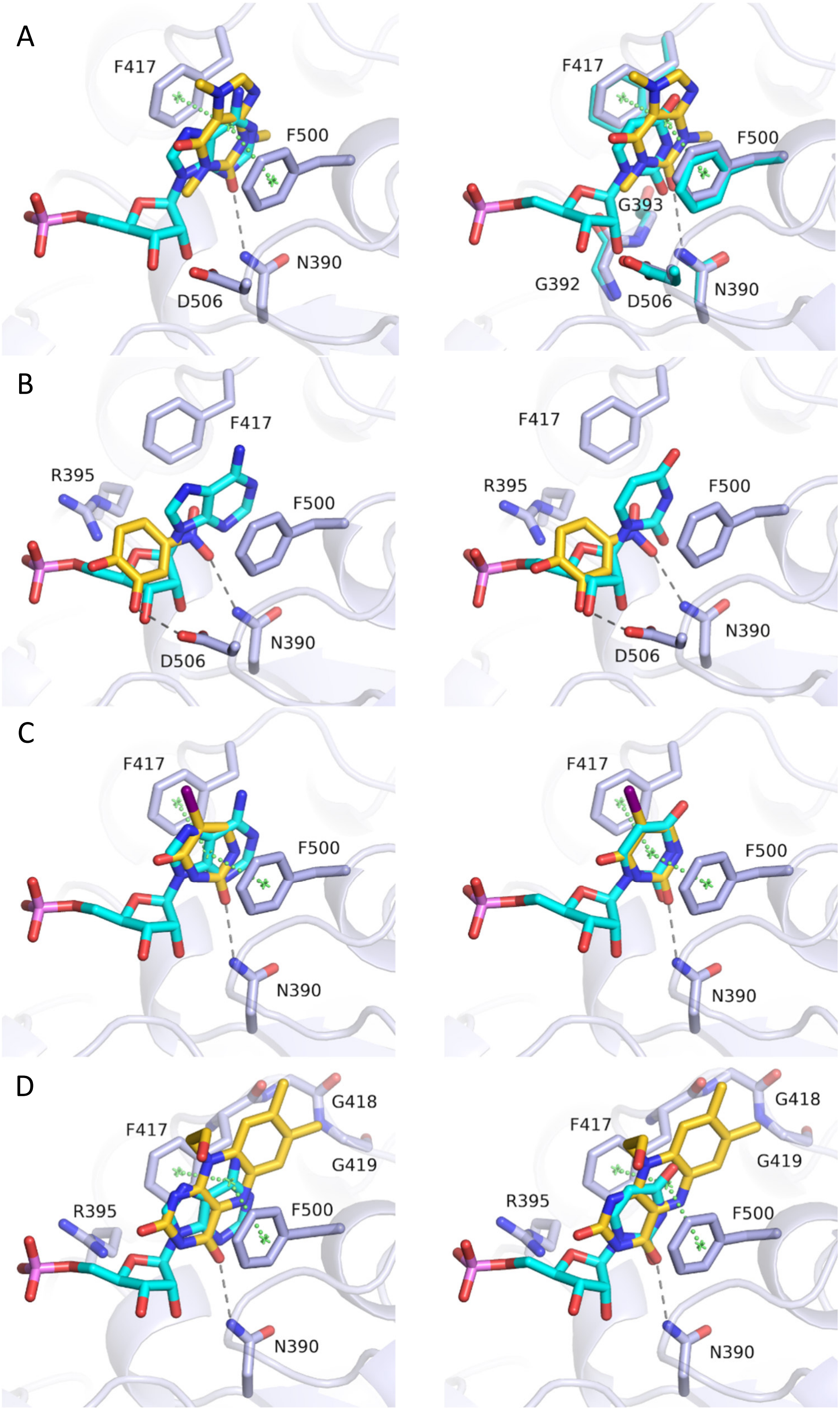

Figure S3: Comparison of the binding modes of four fragments to those of AMP (left side) and UMP (right side). The fragments (A) caffeine, (B) nitrocatechol, (C) iodouracil and (D) riboflavin are shown in yellow and the nucleosides are shown in cyan. The structures are superimposed based on the Cα-atoms of the C-terminal domain.

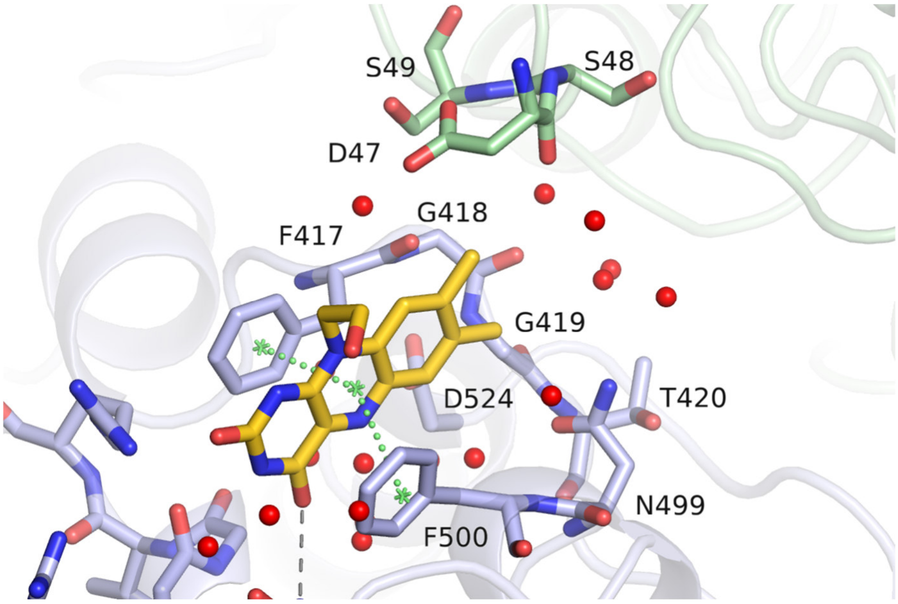

Figure S4: Wider environment of the riboflavin binding site. The flavin group may be extended towards the C-terminal domain and the water-filled interdomain cleft of the open CD73 form to potentially block the enzyme in the inactive open state.

Table S1: Chemical compounds used in this study. Compounds 1-102 of the HZB fragment screen are listed in Table S1 of Huschmann et al. [7].

**Compound**	**Supplier, Product number, purity**
Adenosine 5'-monophosphate (AMP)	Sigma-Aldrich, 01930, ≥ 99 %
2′-Deoxyadenosine 5′-monophosphate (dAMP)	Sigma-Aldrich, D6375, ≥ 98 %
Cytidine 5′-monophosphate (CMP)	Sigma-Aldrich, C1006, ≥ 99 %
2′-Deoxycytidine 5′-monophosphate (dCMP)	Sigma-Aldrich, D7750, ≥ 95%
2′-Deoxyguanosine 5′-monophosphate (dGMP)	Sigma-Aldrich, D9500, ≥ 99 %
2'-Deoxyuridine-5'-[(α,β)-methyleno]diphosphate, Sodium salt (dUMPCP)	Jena Bioscience, NU-428S, ≥ 95 %
Guanosin-5'-monophosphat Dinatriumsalz (GMP)	Sigma-Aldrich, G8377, ≥ 99 %
α,β-Methyleneguanosine 5′-diphosphate sodium salt (GMPCP)	Jena Bioscience, NU-414-5, ≥ 95 %
Adenosin 5'-[α,β-methylene] diphosphoric acid (AMPCP)	Jena Bioscience, NU-420-5, ≥ 95 %
Thymidine 5'-Monophosphate Natriumsalz) dTMP)	Sigma-Aldrich, T7004, ≥ 99 %
Uridin-5'-Monophosphate Dinatriumsalz	Sigma-Aldrich, U6375, ≥ 99 %
Inosine 5'-monophosphate Disodiumsalt	Sigma-Aldrich, U6375, ≥ 98 %
Cytidine-5'-[(α,β)-methyleno]diphosphate, Sodium salt (CMPCP)	Jena Bioscience, I4625, ≥ 95 %

Table S2a. Data collection and refinement statistics

**Data collection**			
Ligand	AMP	GMP	dCMP
PDB ID	7P9N	7P9R	7P9T
Space group	$P2_12_12$	$P2_12_12$	$P2_12_12$
Cell dimensions:			
a, b, c (Å)	67.3, 131.3, 66.3	67.2, 131.5, 66.2	68.1, 132.9, 67.4
Resolution (Å)	47.23-1.55	47.2-1.41	47.9-1.79
Highest shell (Å)	1.58-1.55	1.43-1.41	1.82-1.79
R_{merge} (%)[b]	10.3 (42.2)	7.6 (89.7)	9.1 (87.0)
$CC_{1/2}$	0.997 (0.822)	0.998 (0.551)	0.997 (0.524)
$I / \sigma(I)$	11.5 (2.6)	13.8 (1.5)	11.1 (1.5)
Completeness (%)	92.6 (58.5)	99.8 (98.4)	99.7 (99.1)
Redundancy	6.2 (4.0)	4.1 (3.9)	4.1 (4.1)
**Refinement**			
Resolution (Å)	47.2-1.69	47.2-1.41	48.0-1.79
No. of reflections	62638	107571	55272
R_{work}/R_{free} (%)	15.3/17.8	13.0/16.9	18.5/21.7
No. of atoms:			
Protein	4177	4175	4164
Ligands	32	39	23
Water	479	500	226
B-factors (Å^2)			
Protein	10.9	13.5	17.4
Ligands	11.6	20.2	24.9
Water	22.3	26.9	21.5
R.m.s. deviations:			
Bond length (Å)	0.016	0.020	0.014
Bond angles (°)	2.06	2.02	2.04

*Values in parenthesis are for the highest resolution shell.

Table S2b. Data collection and refinement statistics

**Data collection**			
Ligand	CMP	UMP	IMP
PDB ID	7PA4	7PB5	7PBA
Space group	$P2_12_12$	$P2_12_12$	$P2_12_12$
Cell dimensions:			
a, b, c (Å)	67.6, 131.7, 66.5	67.2, 131.6, 66.2	67.7, 131.9, 66.6
Resolution (Å)	47.4-1.45	47.2-1.28	47.5-1.42
Highest shell (Å)	1.48-1.45	1.30-1.28	1.45-1.42
R_{merge} (%)[b]	7.1 (62.9)	6.8 (58.5)	7.1 (74.1)
$CC_{1/2}$	0.999 (0.600)	0.997 (0.593)	0.999 (0.565)
$I / \sigma(I)$	16.9 (1.9)	10.3 (1.6)	15.9 (1.5)
Completeness (%)	94.3 (64.7)	98.6 (84.8)	99.3 (88.6)
Redundancy	5.9 (3.0)	4.0 (2.8)	6.3 (3.6)
**Refinement**			
Resolution (Å)	47.4-1.45	47.2-1.28	44.7-1.42
No. of reflections	94400	141079	105543
R_{work}/R_{free} (%)	11.9/16.1	12.5/15.7	12.7/16.7
No. of atoms:			
Protein	4183	4175	4221
Ligands	31	66	32
Water	538	468	481
B-factors (Å^2)			
Protein	13.8	13.0	13.2
Ligands	17.0	25.8	14.1
Water	26.1	23.9	24.6
R.m.s. deviations:			
Bond length (Å)	0.015	0.016	0.014
Bond angles (°)	2.02	2.02	1.85

*Values in parenthesis are for the highest resolution shell.

Table S2c. Data collection and refinement statistics

**Data collection**			
Ligand	caffeine	4-nitrocatechol	5-iodouracil
PDB ID	7PBB	7PBY	7PCP
Space group	$P2_12_12$	$P2_12_12$	$P2_12_12$
Cell dimensions:			
a, b, c (Å)	67.2, 131.8, 66.3	67.2, 131.6, 66.4	67.2, 131.8, 66.3
Resolution (Å)	47.2-1.47	47.0-1.13	47.2-1.38
Highest shell (Å)	1.50-1.47	1.15-1.13	1.40-1.38
R_{merge} (%)[b]	17.2 (144.3)	7.5 (107.9)	5.4 (76.8)
$CC_{1/2}$	0.995 (0.348)	0.999 (0.577)	0.999 (0.534)
$I / \sigma(I)$	7.6 (1.4)	11.4 (1.6)	11.3 (1.4)
Completeness (%)	99.0 (95.4)	99.5 (90.2)	99.1 (96.9)
Redundancy	6.6 (6.5)	6.5 (6.0)	3.7 (3.5)
**Refinement**			
Resolution (Å)	47.3-1.47	46.8-1.13	47.3-1.38
No. of reflections	94572	205276	114949
R_{work}/R_{free} (%)	17.1/19.2	12.8/14.3	12.5/16.7
No. of atoms:			
Protein	4218	4242	4257
Ligands	33	46	22
Water	352	500	477
B-factors ($Å^2$)			
Protein	12.6	14.3	17.5
Ligands	21.4	30.0	28.9
Water	20.9	28.7	30.3
R.m.s. deviations:			
Bond length (Å)	0.018	0.012	0.014
Bond angles (°)	2.10	1.76	1.90

*Values in parenthesis are for the highest resolution shell.

Table S2d. Data collection and refinement statistics

**Data collection**	
Ligand	riboflavin
PDB ID	7PD9
Space group	$P2_12_12$
Cell dimensions:	
a, b, c (Å)	67.4, 131.8, 66.5
Resolution (Å)	47.1-1.39
Highest shell (Å)	1.42-1.39
R_{merge} (%)[b]	5.9 (75.1)
$CC_{1/2}$	0.999 (0.700)
$I / \sigma(I)$	13.8 (1.7)
Completeness (%)	98.4 (95.7)
Redundancy	5.0 (5.0)
Resolution (Å)	47.1-1.39
No. of reflections	111357
R_{work}/R_{free} (%)	12.0/14.4
No. of atoms:	
Protein	4206
Ligands	28
Water	516
B-factors (Å^2)	
Protein	15.8
Ligands	20.6
Water	28.3
Bond length (Å)	0.016
Bond angles (°)	1.96

*Values in parenthesis are for the highest resolution shell.

Table S3: List of compounds of the BESSY II fragment screen that bound to the nucleoside binding site of CD73

Fragment	concen-tration (mM)	resolution (Å)	pdb id	remarks
AMPPCP	30	1.48	6TVG[8]	adenosine moiety binds similar to AMP
dGMP	100	1.35	not deposited	similar binding mode of the ribose and phosphate groups as in dCMP
ADP	10	1.53	not deposited	adenosine moiety binds similar to AMP
AMP	100	1.55	7P9N	this work
dCMP	50	1.79	7P9T	this work
NAD		1.40	not deposited	adenosine moiety binds similar to AMP
GMP	10	1.41	7P9R	this work
NADP	100	1.22	not deposited	no density for phosphate group at 2'-OH group of the adenosine moiety visible. Only ADP moiety visible in density.
UMP	10	1.28	7PB5	this work
CMP	100	1.45	7PA4	this work
caffeine	50	1.56	7PBB	this work
4-nitrocatechol	100	1.13	7PBY	this work
IMP	25	1.42	7PBA	this work
dTMP	10	1.38	not deposited	only low occupancy binding
5-iodouracil	10	1.38	7PCP	this work
riboflavin	6.25	1.39	7PD9	this work

References

1. Knapp K, Zebisch M, Pippel J et al. (2012) Crystal structure of the human ecto-5'-nucleotidase (CD73): insights into the regulation of purinergic signaling. Structure 20:2161–2173. https://doi.org/10.1016/j.str.2012.10.001
2. Schliselfeld LH, Burt CT, Labotka RJ (1982) 31P nuclear magnetic resonance of phosphonic acid analogues of adenosine nucleotides as functions of pH and magnesium ion concentration. Biochemistry 21:317–320. https://doi.org/10.1021/bi00531a018
3. Vogel HJ, Bridger WA (1982) Phosphorus-31 nuclear magnetic resonance studies of the methylene and fluoro analogues of adenine nucleotides. Effects of pH and magnesium ion binding. Biochemistry 21:394–401. https://doi.org/10.1021/bi00531a029
4. Bietz S, Urbaczek S, Schulz B et al. (2014) Protoss: a holistic approach to predict tautomers and protonation states in protein-ligand complexes. J Cheminform 6:12. https://doi.org/10.1186/1758-2946-6-12
5. Knöfel T, Sträter N (2001) Mechanism of hydrolysis of phosphate esters by the dimetal center of 5'-nucleotidase based on crystal structures. J Mol Biol 309:239–254. https://doi.org/10.1006/jmbi.2001.4656
6. Sträter N (2006) Ecto-5'-nucleotidase: Structure function relationships. Purinergic Signal 2:343–350. https://doi.org/10.1007/s11302-006-9000-8

7. Huschmann FU, Linnik J, Sparta K et al. (2016) Structures of endothiapepsin-fragment complexes from crystallographic fragment screening using a novel, diverse and affordable 96-compound fragment library. Acta Crystallogr F Struct Biol Commun 72:346–355. https://doi.org/10.1107/S2053230X16004623
8. Bhattarai S, Pippel J, Scaletti E et al. (2020) 2-Substituted α,β-Methylene-ADP Derivatives: Potent Competitive Ecto-5'-nucleotidase (CD73) Inhibitors with Variable Binding Modes. JOURNAL OF MEDICINAL CHEMISTRY 63:2941–2957. https://doi.org/10.1021/acs.jmedchem.9b01611
